# Supplementary material for: Ultrafast all-optical switching in nonlinear 3R-MoS2 van der Waals metasurfaces
Source: Npj Nanophoton. 2025 Sep 2;2(1):37. doi: 10.1038/s44310-025-00083-4 (PMC12404986; doi:10.1038/s44310-025-00083-4)
Supplement: Supplementary file 1 — Supplementary information [file 44310_2025_83_MOESM1_ESM.pdf]

Supplementary information for: Ultrafast all-optical switching in 3R-MoS_2 van der Waals metasurfaces

L. Seidt *et al.*

CONTENTS

Supplementary note I: Quality factors of fabricated metasurfaces 2

Supplementary note II: Numerical modeling of the SHG polarization dependence 2

Supplementary note III: Ultrafast all-optical SHG interference spectra 3

Supplementary note VI: SHG polarization switching in bulk 3R-MoS_2 3

SUPPLEMENTARY NOTE I: QUALITY FACTORS OF FABRICATED METASURFACES

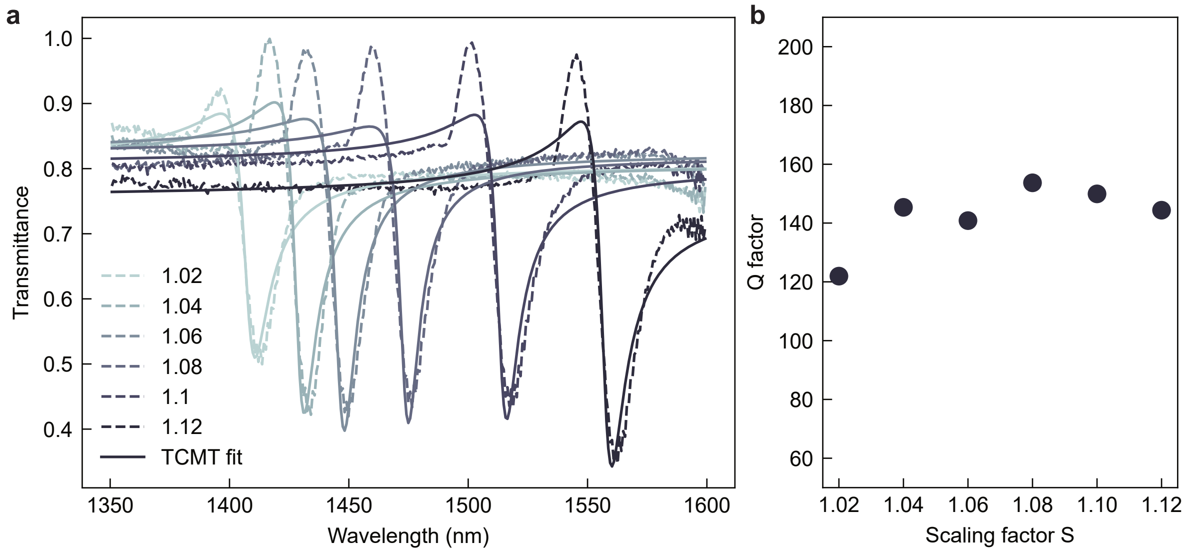

SUPPLEMENTARY FIGURE 1. (a) Experimental transmittance spectra and corresponding TCMT fits. (b) Total quality factors extracted from (a), showing Q factors larger than 100 for all metasurfaces.

SUPPLEMENTARY NOTE II: NUMERICAL MODELING OF THE SHG POLARIZATION DEPENDENCE

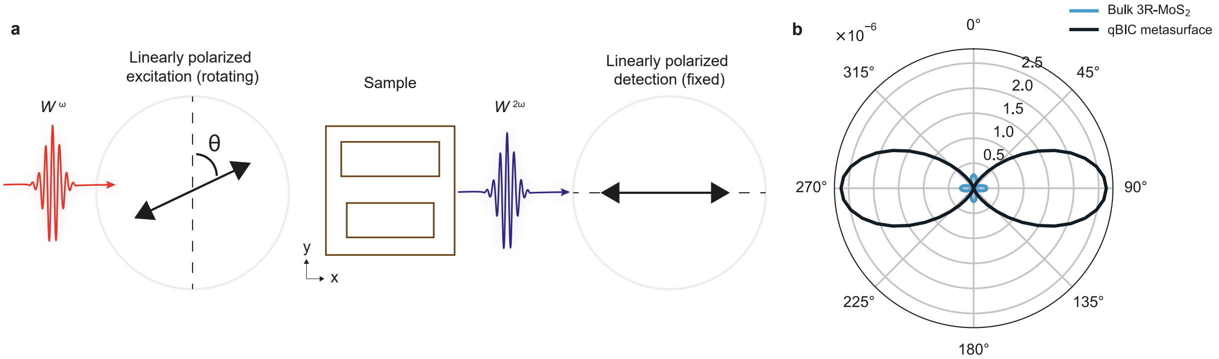

SUPPLEMENTARY FIGURE 2. (a) Schematics of the polarization dependent SHG model. The fundamental beam is rotated respect to the sample, with a fixed linear polarization in the collection. (b) Results of the SHG emission for a 3R-MoS_2 (blue) and a qBIC metasurface (black) of the same thickness.

SUPPLEMENTARY NOTE III: ULTRAFAST ALL-OPTICAL SHG INTERFERENCE SPECTRA

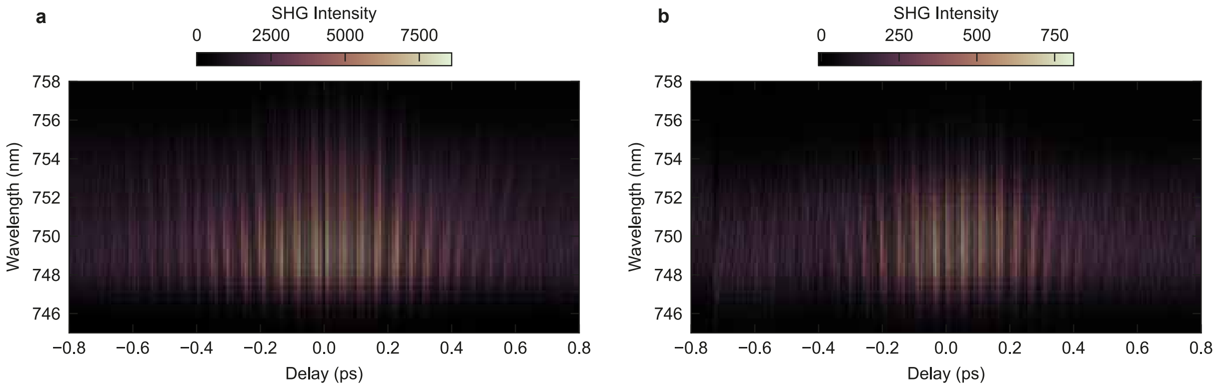

SUPPLEMENTARY FIGURE 3. (a-b) SHG autocorrelation spectra as a function of the time delay between two excitation pulses both aligned along AC, for a 3R-MoS_2 qBIC metasurface. Shown for AC (a) and ZZ (b) detection directions.

SUPPLEMENTARY NOTE VI: SHG POLARIZATION SWITCHING IN BULK 3R-MOS_2

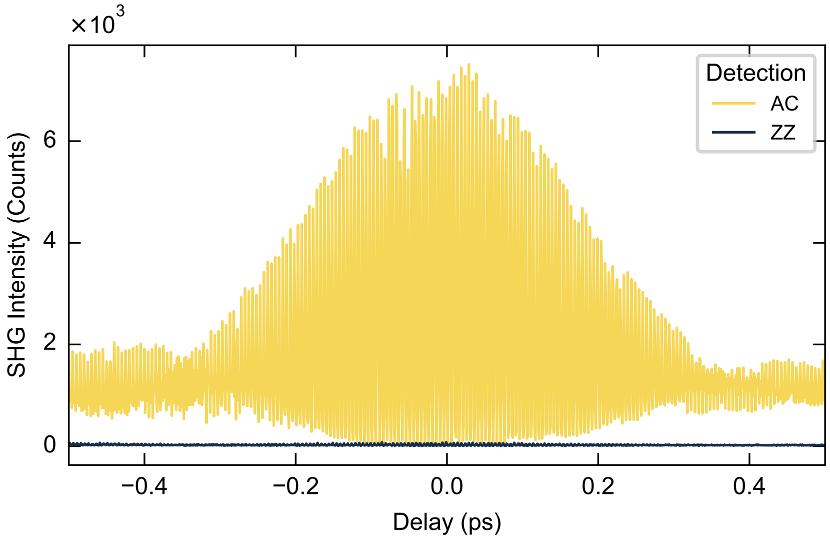

SUPPLEMENTARY FIGURE 4. SHG intensity as a function of the time delay between the two excitation pulses, both polarized along AC, from a bulk unpatterned 3R-MoS_2 sample with thickness of 218 nm. The SHG signal is recorded for linearly polarized detection along AC and for ZZ detection, showing negligible emission in the ZZ direction.
